# Transcriptional Profiling Suggests T Cells Cluster around Neurons Injected with Toxoplasma gondii Proteins

**DOI:** 10.1128/mSphere.00538-20

**Published:** 2020-09-02

**Authors:** Emily F. Merritt, Hannah J. Johnson, Zhee Sheen Wong, Adam S. Buntzman, Austin C. Conklin, Carla M. Cabral, Casey E. Romanoski, Jon P. Boyle, Anita A. Koshy

**Affiliations:** a Department of Immunology, University of Arizona, Tucson, Arizona, USA; b Neuroscience Graduate Interdisciplinary Program, University of Arizona, Tucson, Arizona, USA; c Department of Biological Sciences, Dietrich School of Arts and Sciences, University of Pittsburgh, Pittsburgh, Pennsylvania, USA; d BIO5 Institute, University of Arizona, Tucson, Arizona, USA; e Department of Cellular and Molecular Medicine, University of Arizona, Tucson, Arizona, USA; f Department of Neurology, University of Arizona, Tucson, Arizona, USA; University of Georgia

**Keywords:** RNA-seq, *Toxoplasma gondii*, host-pathogen interactions, immunology, laser capture microdissection, neuroscience, transcriptomics

## Abstract

Toxoplasma gondii’s tropism for and persistence in the central nervous system (CNS) underlies the symptomatic disease that *T. gondii* causes in humans. Our recent work has shown that neurons are the primary CNS cell with which *Toxoplasma* interacts and which it infects *in vivo*. This predilection for neurons suggests that *T. gondii*’s persistence in the CNS depends specifically upon parasite manipulation of the host neurons. Yet, most work on *T. gondii*-host cell interactions has been done *in vitro* and in nonneuronal cells. We address this gap by utilizing our *T. gondii*-Cre system that allows permanent marking and tracking of neurons injected with parasite effector proteins *in vivo*. Using laser capture microdissection (LCM) and RNA sequencing using RNA-seq, we isolated and transcriptionally profiled *T. gondii*-injected neurons (TINs), Bystander neurons (nearby non-*T. gondii*-injected neurons), and neurons from uninfected mice (controls). These profiles show that TIN transcriptomes significantly differ from the transcriptomes of Bystander and control neurons and that much of this difference is driven by increased levels of transcripts from immune cells, especially CD8^+^ T cells and monocytes. These data suggest that when we used LCM to isolate neurons from infected mice, we also picked up fragments of CD8^+^ T cells and monocytes clustering in extreme proximity around TINs and, to a lesser extent, Bystander neurons. In addition, we found that *T. gondii* transcripts were primarily found in the TIN transcriptome, not in the Bystander transcriptome. Collectively, these data suggest that, contrary to common perception, neurons that directly interact with or harbor parasites can be recognized by CD8^+^ T cells.

**IMPORTANCE** Like other persistent intracellular pathogens, Toxoplasma gondii, a protozoan parasite, has evolved to evade the immune system and establish a chronic infection in specific cells and organs, including neurons in the CNS. Understanding *T. gondii*’s persistence in neurons holds the potential to identify novel, curative drug targets. The work presented here offers new insights into the neuron-*T. gondii* interaction *in vivo*. By transcriptionally profiling neurons manipulated by *T. gondii*, we unexpectedly revealed that immune cells, and specifically CD8^+^ T cells, appear to cluster around these neurons, suggesting that CD8^+^ T cells specifically recognize parasite-manipulated neurons. Such a possibility supports evidence from other labs that questions the long-standing dogma that neurons are often persistently infected because they are not directly recognized by immune cells such as CD8^+^ T cells. Collectively, these data suggest we reconsider the broader role of neurons in the context of infection and neuroinflammation.

## Abstract (precis)

Like other persistent intracellular pathogens, Toxoplasma gondii, a protozoan parasite, has evolved to evade the immune system and establish a chronic infection in specific cells and organs, including neurons in the CNS. Understanding *T. gondii*’s persistence in neurons holds the potential to identify novel, curative drug targets. The work presented here offers new insights into the neuron-*T. gondii* interaction *in vivo*. By transcriptionally profiling neurons manipulated by *T. gondii*, we unexpectedly revealed that immune cells, and specifically CD8^+^ T cells, appear to cluster around these neurons, suggesting that CD8^+^ T cells specifically recognize parasite-manipulated neurons. Such a possibility supports evidence from other labs that questions the long-standing dogma that neurons are often persistently infected because they are not directly recognized by immune cells such as CD8^+^ T cells. Collectively, these data suggest we reconsider the broader role of neurons in the context of infection and neuroinflammation.

## INTRODUCTION

Obligate intracellular pathogens are dependent upon host cells for survival. Successful intracellular microbes, therefore, have highly evolved mechanisms to capitalize on host cell resources, avoid clearance by host cell-intrinsic defense mechanisms, and elude the recognition of infected host cells (1, 2). Our understanding of these host cell-microbe interactions primarily comes from *in vitro* studies and/or immune cells, which are relatively easy to isolate (3–6). While such studies form the foundation of our understanding of host-microbe interactions, they have several limitations. These studies most commonly compare infected cultures to uninfected cultures (7–9), which means that some differences assigned to “infection” are likely secondary to paracrine effects on surrounding cells (e.g., interferons). In addition, *in vitro* studies cannot replicate the multifaceted interactions that occur *in vivo*, meaning these studies will miss pathways triggered only during *in vivo* infections. Finally, these studies are often conducted in cells not normally encountered by the pathogen (e.g., fibroblasts), meaning they may miss pathways that are specific to a subset of highly specialized host cells.

Such concerns are highly relevant for a microbe such as Toxoplasma gondii, an obligate intracellular parasite that has a wide range of intermediate hosts including humans and rodents. In most intermediate hosts, T. gondii establishes a persistent, long-term infection in certain organs and cells (10–13). In humans and rodents, the central nervous system (CNS) is a major organ of persistence (14). This persistence and neurotropism underlie the parasite’s ability to reactivate to cause devastating neurologic disease in people with acquired immune deficiencies (e.g., AIDs patients [15–17] or bone marrow transplant patients [18, 19]). Our *in vivo* understanding of CNS toxoplasmosis primarily comes from the mouse model, in which T. gondii preferentially interacts with and persists in neurons (20–23). Thus, neuron-T. gondii interactions likely govern CNS outcomes, including T. gondii persistence. Yet, very little is known about the neuron-T. gondii interaction, especially *in vivo*. What we do know about host cell-T. gondii interactions—that T. gondii secretes many effector proteins into host cells prior to and after full host cell invasion and that these effector proteins can be polymorphic between T. gondii strains, leading to strain-specific host cell manipulations—comes primarily from *in vitro* studies in fibroblasts and immune cells (3, 24–32).

To address this gap in knowledge, we sought to transcriptionally profile two types of neurons from T. gondii-infected mice—those directly manipulated by T. gondii and neighboring neurons that had not been manipulated by T. gondii but were still within in the same cytokine environment (Bystander neurons)—as well as neurons from uninfected mice. We reasoned that such comparisons would allow us to distinguish neuron expression differences arising from direct parasite manipulation from those changes elicited by the general neuroinflammatory response to T. gondii. To accomplish this goal, we utilized our T. gondii Cre system. In this system, Cre reporter mice that express a green fluorescent protein (GFP) only after Cre-mediated recombination (33) are infected with parasites engineered to express a T. gondii:Cre fusion protein that is injected into host cells concomitantly with other early effector proteins and prior to full invasion (34). Thus, T. gondii-injected neurons (TINs) permanently express GFP, while Bystander neurons or neurons from uninfected Cre reporter mice do not, allowing us to use laser capture microdissection to isolate these different groups of neurons. Finally, to enable our ability to identify both universal and strain-specific neuron manipulations, we utilized mice infected with either of the canonical, persistent T. gondii strains. These strains—belonging to either the type II or type III lineage—are genetically distinct, express different polymorphs of some injected effector proteins (24, 25, 27–31), and are known to drive distinct CNS inflammatory responses (32). Thus, with this combination of tools, we sought to define T. gondii’s strain-specific and universal effects on neurons during an *in vivo* infection.

## RESULTS

### Isolation of neurons using laser capture microdissection.

**FIG 1:**
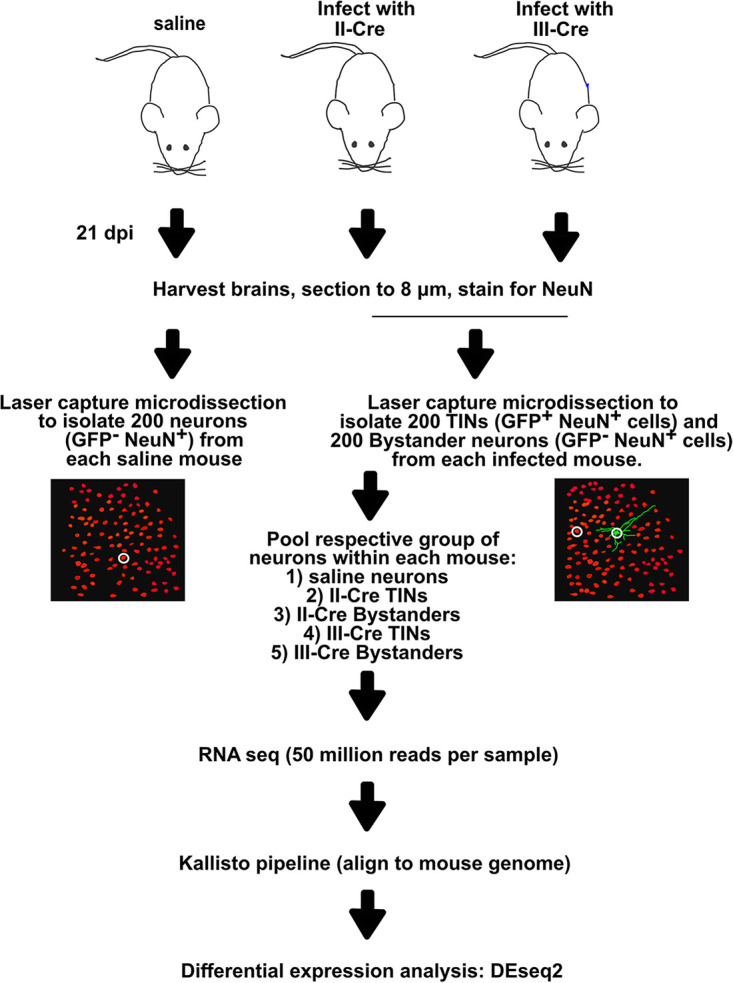
Schematic of experimental design. Mice were intraperitoneally inoculated with either saline or 10k syringe-released II-Cre or III-Cre parasites. At 21 days postinoculation (dpi), brains were harvested, sectioned to 8 μm, and stained for the neuronal marker NeuN. LCM was used to isolate soma of 200 TINs and 200 Bystander neurons from each mouse. Two hundred neurons were isolated from uninfected mice as a control. Each group of samples was pooled, sequenced, and analyzed.

**FIG 2:**
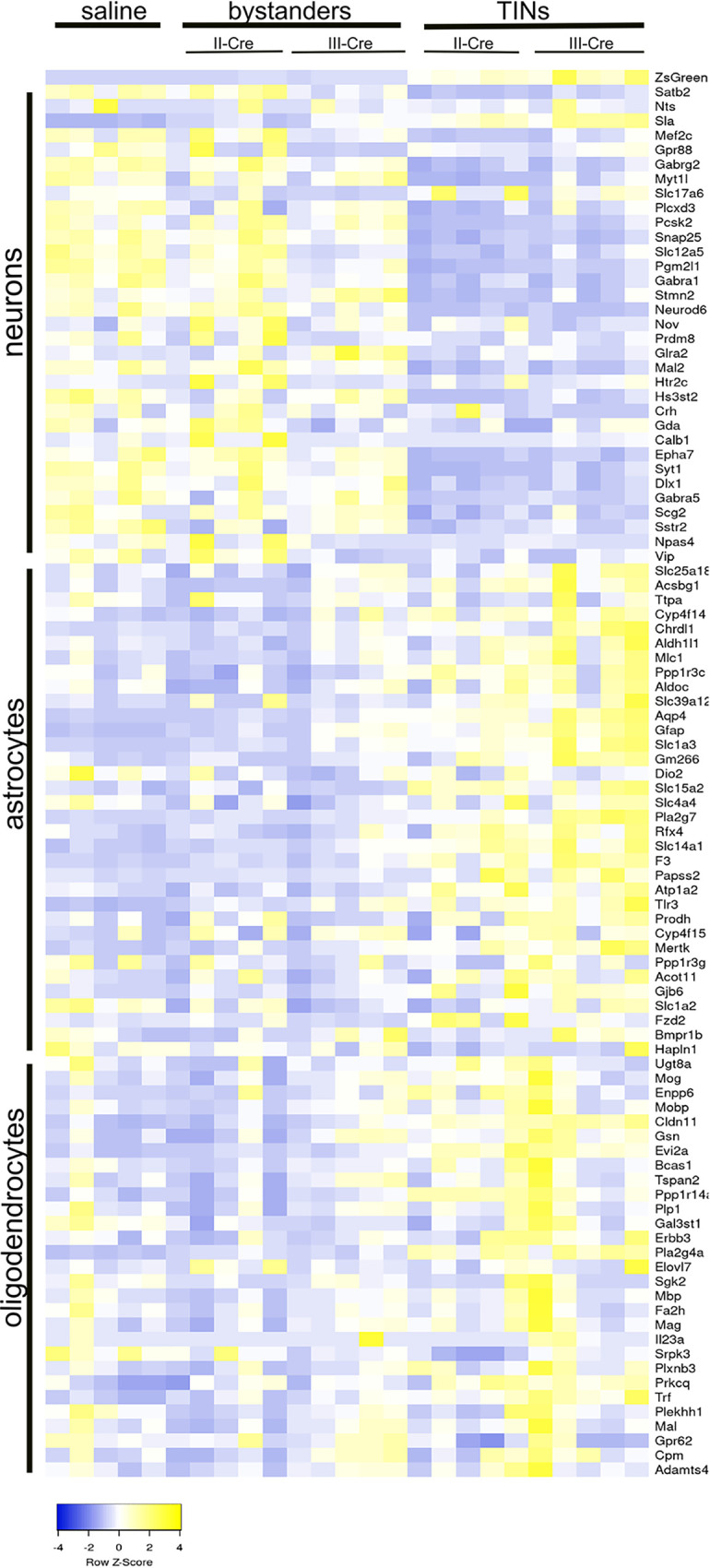
Neuronal transcripts are increased in saline and Bystander samples and decreased in TINs. Five samples for each condition, with 200 neurons per sample, were captured with LCM and sequenced with RNA-seq. Raw reads were evaluated for known neuron-, astrocyte-, and oligodendrocyte-specific transcripts. Heatmap of cell-specific transcript reads; markers curated from the work of Cahoy et al. (37).

To develop insights into how neurons are manipulated by T. gondii *in vivo*, we intraperitoneally inoculated Cre reporter mice (33) with saline (control) or type II (II-Cre) or type III (III-Cre) parasites (Fig. 1). At 21 days postinoculation (dpi), brains were harvested and processed for laser capture microdissection (LCM), including a rapid staining of sections with anti-NeuN antibodies to definitively identify neurons. We then used LCM to isolate 200 cortical neurons (NeuN^+^) from each saline-inoculated mouse, as well as 200 cortical T. gondii-injected neurons (TINs) (GFP^+^ NeuN^+^) and 200 cortical Bystander neurons (GFP^−^ NeuN^+^ and within 80 to 120 μm of a TIN) from each infected mouse (Fig. 1; see also Fig. S1 in the supplemental material). Each 200-neuron group was pooled and used for RNA isolation/library generation. The 25 libraries (5 control, 5 TIN II-Cre, 5 Bystander II-Cre, 5 TIN III-Cre, and 5 Bystander III-Cre) were sequenced at a projected depth of 50 million reads. High-quality reads then underwent pseudoalignment, followed by differential gene expression analysis (35, 36). Our analysis showed that the total number of mapped reads was consistent across samples, with values ranging between 40 and 56 million, except for one III-Cre TIN sample which had approximately 16 million reads (Table S1 and Fig. S2). This sample grouped with the other TINs in principal-component analysis (PCA) (Fig. S2C) and thus was retained through all the subsequent analyses. To validate our isolation technique, we compared the transcript levels of the GFP protein expressed by these mice (ZsGreen) in the saline, Bystander, and TIN samples. As expected, TIN samples showed 42- and 12.5-fold more GFP transcripts than saline and Bystander samples, respectively. Using cell-specific genes identified by Cahoy et al. (37), we compared the enrichment of neuron-specific genes, astrocyte-specific genes, and oligodendrocyte-specific genes within our samples to determine if we had primarily isolated neurons. Cahoy et al. (37) used a rigorous identification method wherein isolated populations of neurons, astrocytes, and oligodendrocytes were separately assessed for cell-type-specific genes that were enriched by a >20-fold change compared to the other CNS cell types. Consistent with primarily isolating neurons, saline and Bystander neurons showed a higher abundance of neuron-specific genes than astrocyte and oligodendrocyte-specific markers (Fig. 2). Unexpectedly, compared to the saline and Bystander neurons, TINs had a decrease in neuron-specific transcripts and an increase in astrocyte- and oligodendrocyte-specific transcripts (Fig. 2).

10.1128/mSphere.00538-20.1 FIG S1 Isolation of soma by laser capture microdissection (LCM). (A) TINs (white arrows) in 8-μm-thick brain section imaged on an Arcturus XT laser capture microdissection system with a Nikon Eclipse Ti-E microscope base. Only white arrows are TINs; residual green stain is due to microscope filters. The microscope uses a mercury lamp and filter cubes that excite (green) 503 nm to 548 nm and (red) 570 nm to 630 nm. We used a NeuN-conjugated antibody to 555 fluorophore. Therefore, when we look in the green channel to visualize TINs, a level of NeuN^+^ 555 antibody is also excited and visualized. In the green channel, the difference between GFP^+^ NeuN^+^ cells and cells that were NeuN^+^ 555 was apparent. Panel 2, NeuN^+^ staining shows neuronal cell body as viewed under a microscope. Panel 3, merged image shows TINs are neurons. (B) Enlarged inset of merged image of panel A. (C) Example of soma isolated by LCM. Panel 1 shows original image. Panel 2 shows brain section after isolation of soma; the area removed is outlined in white. Panel 3 shows soma on cap after isolation. Bleed-through of NeuN^+^ staining is not present in panel C because these are test sections, not stained with NeuN antibody. (D) NeuN^+^ staining of brain section. Panel 1 shows original image. Panel 2 shows brain section after retrieval of soma. Panel 3 shows isolated soma on cap. Download FIG S1, TIF file, 2.8 MB. Copyright © 2020 Merritt et al. 2020 Merritt et al. This content is distributed under the terms of the Creative Commons Attribution 4.0 International license.

10.1128/mSphere.00538-20.2 FIG S2 Reads are almost entirely consistent within and across samples. (A) Mean number or reads per group. (B) Total number of mapped reads per sample. Colors refer to the biological condition of the sample. Reads that map on multiple locations on the transcriptome are counted more than once, as far as they are mapped on less than 50 different loci. Total read counts are similar within and across conditions with IIIT_4 as the one exception, though IIIT_4 still mapped appropriately (see Fig. S4). (C) Rounded counts from Kallisto were analyzed in R Studio. PC1 accounts for 68.62% of the variation, while PC2 accounts for 13.24% of the variation. The TINs are separated from the Bystanders and saline by both principal components, revealing the most variation within the data set. IIIT_4 groups with another type III TIN in transcript variability. Download FIG S2, TIF file, 2.8 MB. Copyright © 2020 Merritt et al. 2020 Merritt et al. This content is distributed under the terms of the Creative Commons Attribution 4.0 International license.

10.1128/mSphere.00538-20.5 TABLE S1 The number of reads per FASTQ RNA-seq file has narrow variation within and across samples. Sample number corresponds to the mouse from which samples were taken. Bystanders and TINs were captured from the same mice. The renamed sample numbers are the names that will often be used throughout figures. Download Table S1, XLSX file, 0.01 MB. Copyright © 2020 Merritt et al. 2020 Merritt et al. This content is distributed under the terms of the Creative Commons Attribution 4.0 International license.

Collectively, these findings show that 200 cells/sample generate high-quality transcriptional data. The high level of neuron-specific transcript in saline neurons and Bystander neurons, in addition to the GFP in TIN transcriptomes, suggests we isolated the cells we sought (cortical neurons, TINs, and Bystander neurons).

### Transcriptomes cluster by T. gondii injection status, not T. gondii strain status.

As the decrease in neuron-specific transcripts in TINs did not segregate by infecting T. gondii strain type, we sought to determine if this segregation by group—but not parasite type—also held true at a global level. To investigate this possibility, we performed a principal-component analysis (Fig. S2C), using all genes with different transcript abundance. Consistent with our findings for parenchymal CNS cell transcripts, saline and Bystander samples clustered together, while TINs were the most distinct group and showed the most within-group variability. Again, neither Bystander nor TIN samples segregated by infecting T. gondii strain (i.e., all TINs cluster together and all Bystanders cluster together), suggesting that global, common differences obscure smaller, strain-specific differences in our data.

**FIG 3:**
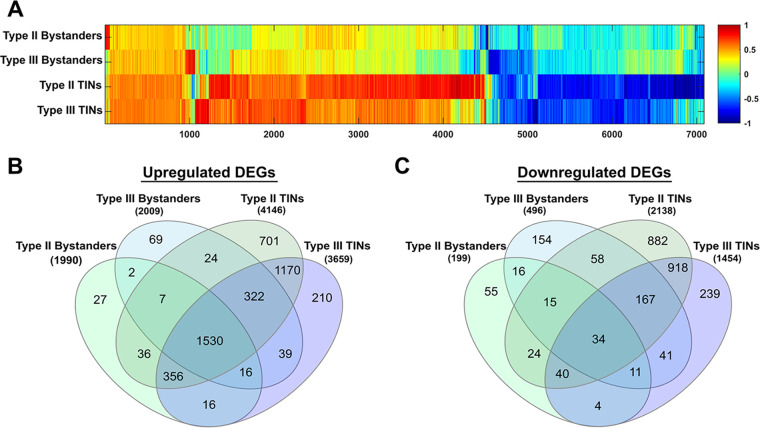
*Toxoplasma*-injected neurons (TINs) cluster together and have the highest number of differentially expressed genes. Comparison of 7,092 genes with ≥2-fold change and with a false-discovery rate of 0.05 at *P*_adj_ of <0.05 across groups, compared to saline controls. (A) Heatmap of 7,092 genes, Bystander and TIN groups normalized to saline with log_2_ fold change; *x* axis indicates number of genes. The log_2_ values of genes were clustered and normalized with the Euclidean norm with the default settings of the MarVis suite. (B) Upregulated DEGs. (C) Downregulated DEGs.

We identified 7,092 genes that differed in our infected groups from saline (Table S2), with marked differences between Bystanders and TINs (Fig. 3A). TINs showed a higher number of unique transcriptional changes (2,081 upregulated, 2,039 downregulated) than did Bystanders (98 upregulated, 225 downregulated) (Fig. 3B and C; Table S2). Collectively, these findings are consistent with prior studies of infected nonneuronal cells (3, 38, 39), indicating that injection with T. gondii effector proteins (and possible infection) causes dramatic alterations in the transcriptional landscape.

10.1128/mSphere.00538-20.6 TABLE S2 Seven thousand ninety-two differentially expressed genes with >2-fold change, with *P*_adj_ of <0.05 for all groups compared to saline controls. Ensembl IDs listed with log_2_ fold values for each condition: type II Bystanders, type II TINs, type III Bystanders, and type III TINs. Additional tabs list upregulated and downregulated DEGs. Download Table S2, XLSX file, 1.6 MB. Copyright © 2020 Merritt et al. 2020 Merritt et al. This content is distributed under the terms of the Creative Commons Attribution 4.0 International license.

### Pathway analysis reveals enrichment for immune pathways in TINs versus Bystanders.

**FIG 4:**
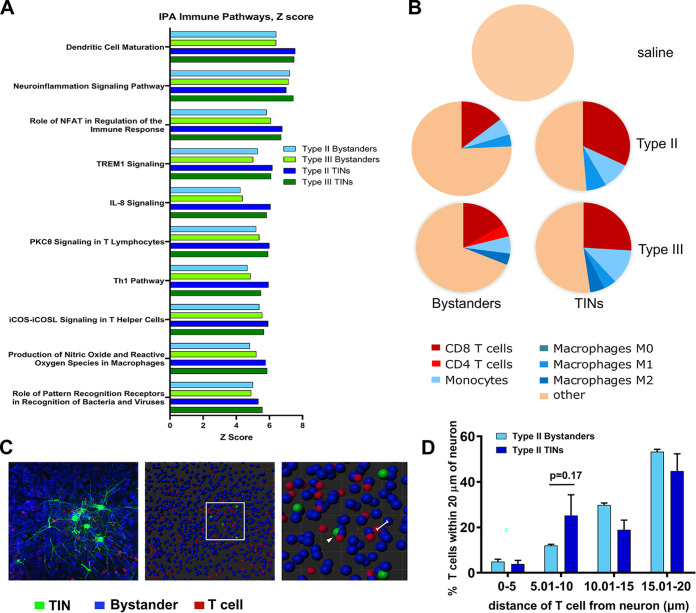
Immune cells cluster around TINs compared to Bystanders and saline. (A) Top 10 immune pathways for type II Bystanders, type III Bystanders, type II TINs, and type III TINs. Z score of upregulation levels of all genes associated with pathways compared to saline. All pathways shown have *P* values of <0.05. (B) Pie charts of CIBERSORT deconvolution using LM22 basis matrix. Each pie chart represents the average proportion of specific immune cell types in the identified samples. Only immune cell types with estimated abundances of ≥4% are shown. Four percent was selected as the cutoff because the average for the saline samples had a *P* value of 1 and all immune cell abundance estimates in the saline samples were <4% (Table S4). The *P* value for the average from all infected deconvolutions was <0.05 (Table S4). Population denoted by “other” represents neurons, astrocytes, oligodendrocytes, microglia, and immune cell estimates that fell below 4%. (C) Sample image of brain section stained with NeuroTrace fluorescent Nissl stain (blue) and anti-CD3ε (red) to visualize Bystander neurons and T cells, respectively. GFP^+^ cells are TINs (green). Panel 2 shows “spots” tool to generate spatial model of cells within image. Panel 3 is enlarged inset of panel 2. The white line shows distance calculation from center to center of each “spot.” The white arrowhead shows T cell “spot” directly adjacent to TIN “spot.” (D) Graph of the percentage of T cells within an identified distance bin. *n* = 4 mice, 2 to 5 images analyzed per mouse. Statistics: 2-way ANOVA.

To investigate the function of the differentially expressed genes (DEGs) in the Bystander neurons and the TINs, we used Ingenuity Pathway Analysis (IPA) to conduct a core analysis on each group and then ran a comparison analysis between groups, all normalized to saline. With no filtering, immune pathways dominated the list (Table S3). To delve into these differences, we filtered our IPA settings to immune and inflammatory pathways. As expected, these pathways were markedly increased in all groups from infected mice compared to the control group (from saline-inoculated mice) (Fig. 4A; Table S3). In most pathways, TINs had higher levels of expression of the genes within a pathway than did Bystanders (Fig. 4A). These data suggested that both Bystander neurons and TINs were in a highly inflamed CNS environment with TINs showing relatively more markers of inflammation.

10.1128/mSphere.00538-20.7 TABLE S3 All canonical pathways listed from IPA for each group compared to saline controls. DEGs from DESeq2 were uploaded to IPA for pathway analysis with either all pathways or immune pathways. Z scores were reported if *P* value was <0.05. This table has been reproduced with the permission of Qiagen. Download Table S3, XLS file, 0.1 MB. Copyright © 2020 Merritt et al. 2020 Merritt et al. This content is distributed under the terms of the Creative Commons Attribution 4.0 International license.

10.1128/mSphere.00538-20.8 TABLE S4 CIBERSORT deconvolution for each sample and immune cell type and microglial reads for each sample. (Tab 1) Each value represents proportion of immune cells contributing to sample. For Fig. 4B, samples were consolidated for clarity of visualization. CD4^+^ T cells include memory, activated, Tfh, Treg, and gamma delta T cells. Average *P* values for infected samples are all <0.05; *P* values for saline samples are 1. (Tab 2) Microglia transcript abundance in saline, Bystander, and TIN samples. Microglia genes were obtained from the work of Bennett et al. (41). Download Table S4, XLSX file, 0.02 MB. Copyright © 2020 Merritt et al. 2020 Merritt et al. This content is distributed under the terms of the Creative Commons Attribution 4.0 International license.

When we examined the upregulated genes in the neuroinflammation pathway, we found many genes that are common in immune responses and produced by many cell types. Such genes include NF-κB, STAT1, major histocompatibility complex class I (MHC I), tumor necrosis factor (TNF), and beta interferon (IFN-β), potentially suggesting that neurons might be mounting typical cellular immune responses to infection and cytokine stimulation (7) and that TINs, the cells with intimate T. gondii contact, received more cytokine signaling and thus generated more robust cytokine responses. But such a hypothesis did not explain the enrichment for immune cell-specific pathways. For example, the Th1 pathway has genes specific to Th1 cells (CD3, CD4, and T cell receptors [TCRs]), which would be highly unlikely to be expressed by neurons (even in the setting of inflammation). This pathway, as well the dendritic cell maturation pathway, suggested that when we used LCM to isolate neurons, we might have also picked up bits of immune cells clustering around Bystander neurons or TINs.

To formally assess this possibility, we analyzed our DEGs with CIBERSORT (40), an algorithm that allows one to estimate the abundance of specified cell types based on gene expression. We performed the deconvolution using the LM22 signature matrix, which can deconvolve a wide range of immune cells, including different macrophage and T cell subsets (e.g., M1 macrophages). Consistent with the lack of inflammation in the brain in uninfected mice, CIBERSORT analysis did not identify significant levels of immune cell transcripts in the saline group (Fig. 4B; Table S4). Of note, the low percentage of various immune cell populations in the saline samples (Table S4) suggests that CIBERSORT detected immune cell transcripts in these samples but that the low transcript abundance was not above the level of noise (*P* value of 1 for these samples). To confirm this interpretation, we compared the top 40 genes specific for microglia (41)—the tissue-resident macrophages of the CNS—in which reads were identified in our samples. We found that the saline samples consistently had low read counts for most of these genes compared to all other samples (Table S4). Collectively, these data highlight that the saline transcriptomes have very little contamination with immune transcripts, including microglia. Conversely, the transcriptomes from infected mice—both Bystander and TIN—showed evidence for moderate-to-high levels of T cell-, monocyte-, and macrophage-specific transcripts. These data are consistent with prior evidence that, during CNS infection with T. gondii, these cell types infiltrate into the CNS and significantly contribute to the control of CNS toxoplasmosis (10, 42–48). The identification of immune cell transcripts within our “neuron” transcriptomes suggests our samples contain transcripts from nonneuronal cells, such as CD8^+^ T cells, that were in extremely close proximity to the isolated neurons.

To assess the possibility that immune cells were in close proximity to TINs and Bystanders and that these transcripts were not a consequence of neurons or other CNS parenchymal cells producing unexpected transcripts in response to an inflammatory environment and infection, we sought to use immunofluorescence to visualize T cells within brain tissue of type II-infected mice. We chose to use type II-infected mice because the transcripts from type II TINs showed the highest level of T cell contamination (Fig. 4B) and recent elegant work by the Blanchard lab has indirectly suggested that CD8^+^ T cells interact with neurons infected with type II parasites *in vivo* (49). To evaluate the distance between T cells and TINs or Bystander neurons, 200-μm-thick brain sections from type II-infected mice were optically cleared and stained using anti-CD3ε antibodies and NeuroTrace fluorescent Nissl stain to identify T cells and neurons, respectively (Fig. 4C, panel 1). Stained sections were then imaged by confocal microscopy, after which the resultant images were analyzed using the “spots” tool in Bitplane Imaris software. This tool creates a rendering of the image in which the software uses the different fluorescent stains to place a “spot” where each neuron cell body or T cell is present. We then generated the distances of T cells to each TIN and Bystander in the field of view by measuring the center-to- center distance between appropriate “spots” (Fig. 4C, panels 2 and 3). As we were interested in T cells with extreme proximity to neurons, we restricted our analysis to T cells that were within 20 μm of each TIN or Bystander within our image. For these T cells, we binned the percentage that were within 0 to 5 μm, 5.01 to 10 μm, 10.01 to 15 μm, and 15.01 to 20 μm of a TIN or Bystander. Because these distances are generated center to center, the 0- to 5-μm distance would generally be expected to fall within a single cell (neuron cell bodies are ∼10 μm). T cells within the 5.01- to 10-μm bin would be consistent with a T cell in direct apposition to a neuron. Such proximity would be required for T cell receptor-major histocompatibility complex I (MHC I) interactions. A higher percentage of T cells fell into the 5.01- to 10-μm bin for TINs than for Bystanders, though this difference was not statistically significant (Fig. 4D) (*P* = 0.17, 2-way analysis of variance [ANOVA]).

Collectively, these data are consistent with prior work showing upregulated inflammatory pathways and an increase in immune cell infiltration in the CNS during infection (49–52). Our identification of immune cell transcripts within our “neuron” transcriptomes and visualization of CD3^+^ T cells suggest our samples contain transcripts from nonneuronal cells, such as CD8^+^ T cells, that may be in extreme proximity to the isolated neurons.

### Parasite transcripts are primarily found in the TIN transcriptome.

**FIG 5:**
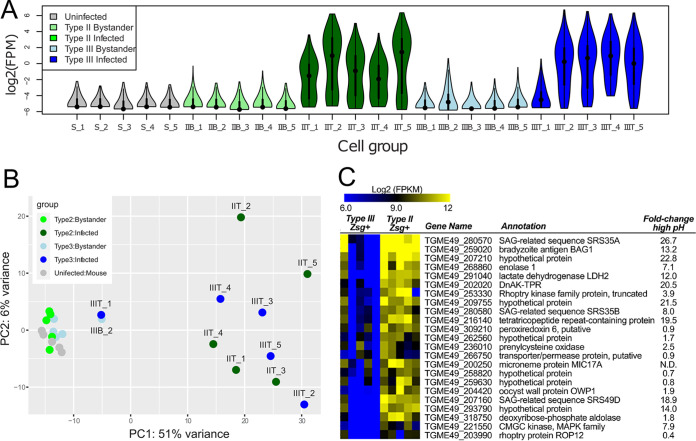
T. gondii bradyzoite transcripts have a higher abundance in type II TIN transcriptomes. (A) Violin plot showing normalized [log_2_(FPM)] read counts for T. gondii genes across samples. These graphs show data for 527 genes selected based on having fewer than 20 mapping reads across the 5 saline-injected mouse control samples and at least 1 read in a minimum of 15 of the 25 samples. (B) Principal-component analysis of the data shown in panel A, illustrating general clustering between samples from Bystander cells and saline-inoculated controls, and separation between TINs and all other samples. (C) Log_2_(FPM) values for a cluster of genes with distinct transcript abundance between type II and type III TINs. This cluster includes multiple canonical bradyzoite markers including BAG1, DnAK-TPR, and LDH2. In addition, most genes in this cluster are known to increase in abundance after high-pH exposure in multiple T. gondii strain types, including the type III strain VEG (53).

Given that our TIN transcriptome contained a higher number of transcripts from immune cells than did our Bystanders, we sought to determine how parasite transcripts partitioned between TINs and Bystanders. After eliminating all reads that mapped to the host transcriptome (see Materials and Methods), we mapped the remaining reads against the T. gondii genome and quantified putative parasite transcript abundance in all samples (including neurons from saline-injected controls). We first eliminated all genes that had more than 20 total reads across all 5 saline-injected samples (as any significant mapping to the parasite transcriptome in these samples would represent genes with high similarity between parasite and host), leaving 8,605 transcripts that we analyzed in a number of ways. To qualitatively assess the quantity of parasite transcripts in TINs versus Bystanders, we further filtered the data to include only genes with at least 1 read in 15 of the samples, leaving 527 genes, for which we calculated log_2_-transformed fragments per million (FPM) reads mapped to the host transcriptome (Table S5). We did this to normalize each sample independently of the number of mapping parasite reads so that the samples could be compared independently of infection status and to use only genes of comparatively high abundance in the TINs. The TIN cell populations had consistently higher read counts for parasite transcripts than did Bystander cells, with the exception of sample IIIT_1 (Fig. 5A). This finding was also reflected in the PCA (Fig. 5B), where all TIN samples except IIIT_1 clustered together along the major PC1 axis, while all Bystander samples except IIIB_2 clustered along this same axis with saline-injected control samples (unlabeled cluster on left, Fig. 5B). These data show that, as expected, TINs harbor the majority of parasite transcripts sequenced from LCM-captured cells.

10.1128/mSphere.00538-20.9 TABLE S5 T. gondii transcript abundance in TINs, type II versus type III. List of T. gondii transcripts that were significantly upregulated compared to saline control and then normalized between strains (type II versus type III) to show relative abundance between II-Cre TINs and III-Cre TINs. The comparison is visualized in Fig. S3. Download Table S5, XLSX file, 0.4 MB. Copyright © 2020 Merritt et al. 2020 Merritt et al. This content is distributed under the terms of the Creative Commons Attribution 4.0 International license.

To look at only the infected cells in isolation from the Bystander and cells from uninfected mice (saline controls), we first selected all genes with at least 1 read in at least 6 of the 25 samples (leaving 1,927 genes) and used DESeq2 to normalize and transform the data. We performed cluster analysis and found that most genes were similarly expressed between strains (Fig. S3A). While few genes were found to be significantly different in abundance between strains (likely due to the high variance in transcript counts across samples), we did identify a cluster of genes that were consistently of higher transcript abundance in type II TINs than in type III TINs (Fig. 5C). Genes within this cluster included multiple canonical markers for the bradyzoite life stage, including BAG1, LDH2, and DnAK-TPR (Fig. 5C and Fig. S3), suggesting a possible difference in bradyzoite status between type II and type III strains at 21 dpi when these samples were collected. To further explore these data, we compared the cluster of T. gondii genes with higher abundance in type II TIN versus type III TIN transcriptomes with published transcript abundance from a different type III strain (VEG) after bradyzoite conversion via high pH (Fig. 5C; data taken from the work of Sokol et al. [53]). In this comparison, we found that many of the genes in the higher-in-type-II TIN cluster were also induced in VEG bradyzoites compared to tachyzoites. When we performed gene set enrichment analysis (GSEA) (54) using the previously published data (53) to create “bradyzoite” and “tachyzoite” gene sets, we found significant enrichment of the bradyzoite gene set in the type II TIN transcriptome and the tachyzoite gene set in the type III TIN transcriptome (Fig. S3B). Overall, these data are consistent with a difference in bradyzoite transcript abundance, and possibly developmental status, between type II and III strains at this time point.

10.1128/mSphere.00538-20.3 FIG S3 Type II and type III T. gondii strains differ in bradyzoite and tachyzoite gene expression. (A) Plot comparing log_2_(FPM) values for 1,923 T. gondii transcripts identified for type II and type III TINs. Data were selected if reads could be found in at least 6 of the 25 samples. Error bars represent standard errors of the means (*n* = 5 for each strain type), and data points are colored red if they had at least a log_2_ difference of 2 between type II or type III TINs. (B) Gene set enrichment analysis of 1,923 transcripts ranked by transcript abundance in type II versus type III TINs compared to a curated bradyzoite (left) and tachyzoite (right) gene set. Type II parasites have significant enrichment for the BZ gene set while type III parasites have significant enrichment for the TZ gene set. Download FIG S3, TIF file, 2.7 MB. Copyright © 2020 Merritt et al. 2020 Merritt et al. This content is distributed under the terms of the Creative Commons Attribution 4.0 International license.

## DISCUSSION

Here, we sought to gain the first insights into how T. gondii parasites manipulate neurons *in vivo* by leveraging a mouse model in which parasites trigger host cell green fluorescent protein (GFP) expression (34, 55) in combination with laser capture microdissection and transcriptional analysis. We chose to use LCM to isolate both T. gondii-injected neurons (TINs) and nearby uninjected neurons (Bystanders) to control for paracrine cytokine effects that do not occur in uninfected mice. Using this technique, we generated high-quality host and parasite transcripts that showed robust group-specific transcriptional profiles (Fig. 3A; see also Fig. S2 in the supplemental material) despite the relatively small sample size (200 cells/group). Unexpectedly, the separation between the groups, including between TINs and Bystanders, was driven primarily by differences in immune cell genes rather than differences in neuron signaling pathways (Fig. 4A). Further analysis suggested that immune cell clustering around TINs, and, to a lesser degree, Bystander neurons, explained much of the group-specific differences (Fig. 4B to D). In addition, a complex parasite transcriptional profile was detected in TINs and this profile was notably absent from Bystander transcriptomes. Collectively, these data suggest that at least a subpopulation of the TINs that we collected were infected with T. gondii parasites (in addition to being injected) and that immune cells, especially CD8 T cells, hone in on TINs.

**FIG 6:**
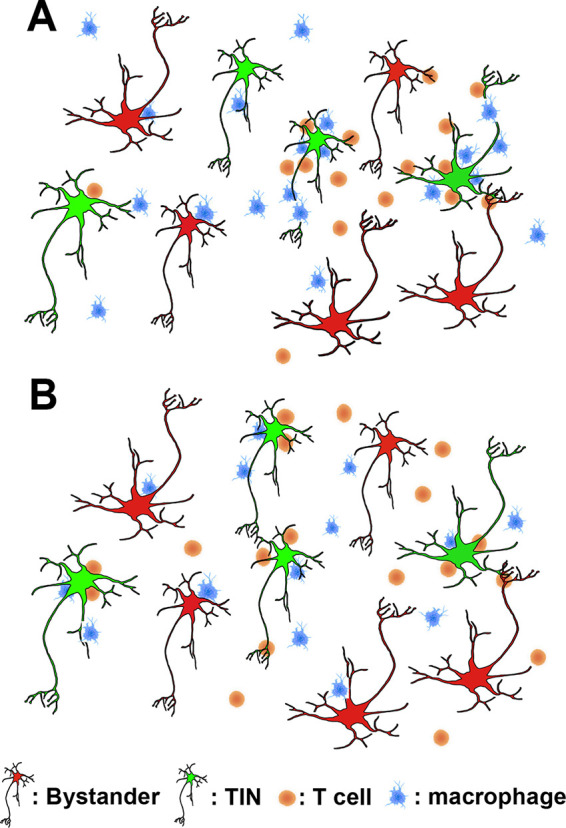
Proposed models of immune cell clustering around TINs. (A) Model of macrophages and T cells clustering around select TINs. (B) Model of uniform clustering of macrophages and T cells around TINs.

Why is our identification of immune cell transcripts in the T. gondii-infected brain novel? All prior genome-wide expression studies of the T. gondii-infected CNS have also identified high levels of immune cell or immune response transcripts compared to uninfected brain (50–52). But these studies have all been done using whole brain for RNA isolation, where one would expect to obtain transcripts from all cells within the brain (parenchymal CNS cells and infiltrating immune cells). Conversely, we tried to avoid nonneuronal cells by using laser capture microdissection to isolate 10-μm-diameter areas centered on neuron somas, which are ∼10 μm in diameter. In addition, instead of simply comparing our TIN transcriptomes to transcriptomes derived from uninfected mice, we isolated Bystander neurons which are 8 to 12 cell bodies away from an isolated TIN. The enrichment for immune cell transcripts in the TIN transcriptomes compared to transcriptomes from uninfected mice and Bystander neurons indicates that these immune cells, especially CD8 T cells, may be in close proximity to TINs, suggesting the T cells may “recognize” the TINs. The potential recognition of infected or injected neurons by T cells supports a notable shift in our understanding of neuron capabilities for generating cellular immune responses. Only in the last several decades have neurons been shown to express MHC I at baseline and, *in vitro*, to have the capability to stimulate CD8^+^ T cells (56–59). An increase in T cell transcripts from bits of immune cells being captured in close proximity to LCM-captured TINs compared to Bystanders, in conjunction with the presence of T. gondii transcripts in TINs, further strengthens the possibility of an interaction between neurons, T. gondii, and T cells. One caveat is that because our individually isolated cells were pooled (i.e., all 200 TINs from a single mouse were collected together after which RNA was isolated), we cannot distinguish whether the immune cell signatures arose equally from each TIN or if a subset of TINs had much higher numbers of aggregated immune cells (Fig. 6). Such clustering of T cells around very specific TINs could explain why our imaging analyses only showed a trend for T cells to cluster more with TINs than with Bystanders. It is also possible that T cells cluster equally around TINs and Bystanders but that the ones near TINs are more activated and thus express a larger amount of “activated” CD8^+^ T cell transcripts than do T cells around Bystanders. While future work will focus on distinguishing between these possibilities, any of these models suggests that the TIN-immune cell interaction is distinct from the Bystander-immune cell interaction, adding to the growing literature that, contrary to dogma, infected neurons have robust immune responses to microbes and cytokines and can present antigens to T cells *in vivo* (7, 49, 60).

One complication of the high abundance of immune transcripts is that we were unable to accomplish our original goal of defining T. gondii strain-specific manipulations of neurons. When normalized to saline, the top genes in type II TINs and type III TINs are almost identical and largely composed of immune response transcripts (Fig. S4). Even when we compare only type III TINs to type II TINs, which leads to strain-specific separation of samples on PCA, we are unable to distinguish what transcripts are inherent to neurons and what transcripts are from clustering immune cells (Fig. S4). Thus, future work to define T. gondii strain-specific differences in neuron signaling will require a technique in which neurons can be cleanly separated from the infiltrating immune cells, such as brain digestion followed by cell sorting. Such a technique was used in the only other study that has sought to identify how microbe-manipulated neurons are affected *in vivo* (61). This study used an attenuated rabies virus (RBV)-Cre system in combination with flow cytometry to isolate previously infected neurons at 6 months postinfection (61). While that study was able to successfully separate out RBV-affected neuron transcripts, as the uninfected, Bystander neurons came from throughout the mouse brain, the study could not control for paracrine changes versus direct RBV-induced changes. Nor could the study be used to identify RBV transcripts as the time point assessed was well beyond when the virus was cleared.

10.1128/mSphere.00538-20.4 FIG S4 The most upregulated transcripts in type II and type III TINs are immune related. DEGs from DESeq2 for II-Cre TINs and III-Cre TINs normalized to saline were analyzed in R Studio. (A and B) The top 12 genes are highlighted in green for II-Cre TINs (A) and blue for III-Cre TINs (B). Many of the same genes are expressed in both groups and are immunity related. DEGs from DESeq2, with III-Cre TINs normalized to II-Cre TINs, reveal that differences in transcripts are possibly due to immune cells. (C) Volcano plot of type III TINs normalized to type II TINs. As an example, S100A5, downregulated in III-Cre TINs compared to II-Cre TINs in the upper left corner, may be from infiltrating immune cells, not neurons. (D) PCA plot of TINs. Principal component 2 (PC2) separates TINs by strain. Type II TINs cluster to the top of the graph while type III TINs are only in the bottom half of the graph. Download FIG S4, TIF file, 2.8 MB. Copyright © 2020 Merritt et al. 2020 Merritt et al. This content is distributed under the terms of the Creative Commons Attribution 4.0 International license.

Conversely, we were able to identify parasite transcripts in the TIN transcriptomes (Fig. 5A). Akin to the immune cell transcripts, several models could account for the parasite transcripts being in only the TIN transcriptomes. As recent papers have shown that uninfected-injected host cells have different transcriptional profiles than fully invaded cells and that these differences are driven by the parasite effectors that are released before full invasion (rhoptry proteins) versus after invasion (specific dense granule proteins) (39, 62), follow-up studies using single-cell RNA-seq should be able to identify if an individual TIN arose from aborted invasion or from invasion followed by clearance of the intracellular parasite. Such studies may even be able to distinguish between previously infected neurons and actively infected neurons.

Regardless of the origins of the transcript, the strain-specific differences in developmental transcripts—a higher abundance of bradyzoite genes in type II TINs versus a higher abundance of tachyzoite transcripts in type III TINs (Fig. 5C; Fig. S3)—are intriguing. These data suggest that type II and type III strains may have different rates of conversion from tachyzoites to bradyzoites *in vivo*, with the type III strain trailing behind the type II strain at the 21-dpi time point, despite the fact that type III strains are very capable of forming cysts *in vivo* (23, 32, 63). We recently described how the host immune response at the same time point differs between these strains, even though the CNS parasite burden was equivalent by Q-PCR for a T. gondii-specific gene and cyst counts (32). The transcriptional data presented here suggest that, *in vivo*, type II and type III parasites differ in their rate of stage conversion, leading to a proverbial chicken-or-the-egg question. Do strain-specific differences in host immune signals dictate the rate of parasite conversion upon arrival to the brain? Or do differences in the intrinsic rates of parasite stage conversion in the CNS lead to strain-specific immune responses? Or both? Ultimately, these data suggest that how T. gondii successfully establishes a persistent CNS infection may vary by strain type.

In summary, the data presented here suggest that neurons injected with T. gondii proteins are surrounded by infiltrating immune cells and that, *in vivo*, T. gondii strains may differ in the rate in which they convert from the lytic tachyzoite form to the persistent bradyzoite form. Collectively, these data suggest we have much to learn about neuron-parasite-immune cell interactions and how these interactions vary by T. gondii strain.

## MATERIALS AND METHODS

### Ethics statement.

All mouse studies and breeding were carried out in strict accordance with the Public Health Service Policy on Human Care and Use of Laboratory Animals. The protocol was approved by the University of Arizona Institutional Animal Care and Use Committee (no. A-3248-01, protocol no. 12–391).

### Mice.

All mice used in this study are Cre reporter mice that express GFP in their cells only after Cre-mediated recombination (33). Mice were purchased from Jackson Laboratories (stock no. 007906) and bred in the University of Arizona BIO5 Animal Facility. Mice were inoculated intraperitoneally (i.p.) with freshly syringe-released parasites, diluted to the appropriate inocula in a 200-μl volume in USP-grade phosphate-buffered saline (PBS). The inoculating number of parasites was 10,000 for II-Cre and III-Cre.

### Parasites.

All strains were maintained through serial passage in human foreskin fibroblasts (gift from John Boothroyd, Stanford University, Stanford, CA) using Dulbecco modified Eagle medium (DMEM), supplemented with 10% fetal bovine serum, 2 mM glutagro, and 100 IU/ml penicillin/100 μg/ml streptomycin. Type II, Prugniaud, referred to as II-Cre, and type III, CEP, referred to as III-Cre, strains express Cre and mCherry (34).

### LCM brain tissue preparation.

Cre reporter mice were infected with an inoculum of 10,000 parasites for 21 days. Upon harvest, brains were removed and cut into 2 hemispheres. Brains were washed in sterile PBS containing ProtectRNA (Sigma-Aldrich; R7397-30ML), moved to a dish containing sterile 4% paraformaldehyde (PFA) for 5 to 10 min, and then transferred to another dish containing sterile PBS containing ProtectRNA. Hemispheres were then flash frozen in O.C.T. and isopentane and sectioned to 8 μm on a cryostat.

### LCM brain tissue staining.

Mounted sections were quickly thawed and dipped in nuclease-free water and ProtectRNA (RNase inhibitor; Sigma-Aldrich; R7397-30ML) several times to remove O.C.T. Excess water was removed from the slides, and 100 to 200 μl antibody solution was added to the sections (anti-NeuN antibody, clone A60, Alexa Fluor 555 conjugate-MAB377A5 [1:200]) and placed on a cold plate on ice, covered, for 10 min. Excess antibody was wicked away, and sections were washed two times with sterile PBS and ProtectRNA for 10 s. Samples were dehydrated by consecutively submerging slides in 70% ethyl alcohol (EtOH) for 60 s, 95% EtOH for 60 s, and 100% EtOH for 60 s. The 100% EtOH step was repeated two times, followed by two 60-s washes in xylenes (64). Samples were air dried for 5 min, and LCM was performed immediately.

### LCM.

Laser capture microdissection was performed on an Arcturus XT laser capture microdissection system with a Nikon Eclipse Ti-E microscope base. The Arcturus XT epifluorescent illumination package utilizes custom filter cubes: green, excitation 503 nm to 548 nm, emission >565 nm; red, excitation 570 nm to 630 nm, emission >655 nm; and triple dichroic cube (4′,6-diamidino-2-phenylindole [DAPI]/fluorescein isothiocyanate [FITC]/tetramethyl rhodamine isocyanate [TRITC]) (part no. 6530-0056), excitation 385 to 400/475 to 493/545 to 565 nm, emission 450 to 465/503 to 533/582 to 622 nm.

### RNA isolation, preparation of cDNA libraries, and sequencing.

RNA samples were assessed for quality with an Advanced Analytics fragment analyzer (high-sensitivity RNA analysis kit no. DNF-491) and quantity with a Qubit RNA quantification kit (Qubit RNA HS assay kit no. Q32855). Samples were used for library builds with the Clontech Smart-Seq V4 Ultra Low Input RNA kit from TaKaRa (catalog no. 634890). Upon library build completion, samples had quality and average fragment size assessed with the Advanced Analytics fragment analyzer (high-sensitivity NGS analysis kit no. DNF-486). Quantity was assessed with an Illumina universal adaptor-specific qPCR kit from Kapa Biosystems (Kapa library quantification kit for Illumina NGS no. KK4824). After final library quality control (QC) was completed, samples were equimolar pooled and clustered for sequencing on the NextSeq500 machine. The paired-end sequencing run was performed using Illumina HighSeq2500 run chemistry (HiSeq2500 high-output 2x100PE 8-lane run, 200 total cycles, FC-401-3001).

### Data processing and differential gene expression analysis, host transcripts.

An average of 57.7 million raw reads per library were obtained for 25 distinct barcoded Illumina RNA-seq libraries (5 control, 5 TIN II-Cre, 5 Bystander II-Cre, 5 TIN III-Cre, and 5 Bystander III-Cre). To prepare the raw data for differential gene expression analysis, the reads from the Illumina FASTQ files from each Illumina library were pseudoaligned to the Mus musculus reference transcriptome (Ensembl GRCm38.81) and a determination of transcript abundance was performed with Kallisto, which utilizes a combination of k-mer hashing and a transcriptome de Bruijn graph for accurate pseudoalignment (36). The transcript-level abundance measures that were determined by Kallisto were converted to length-scaled gene-level abundances with tximport and reported in units of transcripts per million reads (TPM) (65). The length-scaled gene-level abundances were then analyzed for differential gene expression between the 5 analysis groups utilizing DESeq2 (35). Tximport and DESeq2 analysis were performed on the CyVerse infrastructure (66).

The analysis was performed using the R software (67), Bioconductor (68) packages including DESeq2 (35, 69), and the SARTools package developed at PF2-Institut Pasteur. Normalization and differential analysis are carried out according to the DESeq2 model and package. Briefly, the raw data were reported with mapped reads per group, the proportion of null reads not included in further analysis, and the distribution of reads across groups which were very similar. To assess the similarity between samples within replicates and across conditions, the simple error ratio estimate (SERE) statistic was used to measure whether the variability between samples is random Poisson variability or higher (70). DESeq2 then transforms the data to account for differential variance across range of the means to render data that approach homoscedasticity, using the variance stabilizing transformation (VST) (35, 69) method as shown in cluster dendrogram and principal-component (PC) plots. DESeq2 then normalized the data by computing a scalar factor for each sample, assuming that most of the genes will not be differentially expressed, with the default setting locfunc = “median.” The differential analysis performed by DESeq2 fits one linear model per feature with log_2_(FC [fold change]) that calculates *P* and *q* values. Outliers were calculated by Cook’s distance (71) and were not assigned a *P* value. The dispersion estimate was set to the default setting of generalized linear model (GLM). Then, DESeq2 imposed a Cox Reid-adjusted profile likelihood maximization (72, 73) and used the maximum *a posteriori* (MAP) of the dispersion (74). DESeq2 finally plotted the raw *P* values for differential expression and performed independent filtering to increase detection power (35). For the final results, including an adjusted *P* value calculation, a Benjamini-Hochberg (BH) *P* value adjustment was performed (75, 76) and the level of controlled false-positive rate was set to 0.05.

### Pathway analysis.

Differentially expressed genes were uploaded into Ingenuity Pathway Analysis (Qiagen), with one set normalized to saline as well as TIN groups normalized to their respective Bystanders (type II TINs normalized to type II Bystanders, etc.). A “Core Analysis” was run on each group before a “Comparison Analysis” was run between groups. “Canonical Pathways” and “Diseases and Functions” analysis was performed for inflammatory and immune signals with the following filter settings: canonical pathways → Filter → Cellular Immune Response, Cytokine Signaling (T Helper Cell Differentiation, Th1 Pathway, Th1 and Th2 Activation Pathways, Th2 Pathway), Humoral Immune Response, and Pathogen-Influenced Signaling. Diseases and Functions → Filter → Diseases and Disorders (Antimicrobial Response), Molecular and Cellular Functions (Cell-to-Cell Signaling, Cellular Function and Maintenance, and Cellular Movement), and Molecular and Cellular Functions, Physiological System Development and Function.

### CIBERSORT analysis.

Normalized gene expression values for uninfected, Bystander, and TIN samples and the LM22 signature matrix were used as input to CIBERSORT (40). The deconvolution was performed on Stanford University’s online CIBERSORT webpage tool. Both absolute and relative modes were run, and quantile normalization was disabled. One thousand permutations were run for statistical testing. Saline samples had *P* values = 1, suggesting that small levels (<4%) of immune cell abundance in the saline samples were not significant. For this reason, for samples with *P* values of <0.05, only immune cell populations above the saline-based threshold of 4% are shown.

### T cell stain and distance analysis using Imaris software.

Cre reporter mice were inoculated with II-Cre as described above. At 21 dpi, mice were perfused with cold PBS followed by 4% PFA and drop fixed in 4% PFA overnight before being transferred to 30% sucrose solution. Brains were sectioned to 200 μm on a vibratome and processed using the PACT clearing technique described elsewhere (77). After clearing, using the previously described microwave protocol (78), brain sections were stained with anti-CD3ε antibodies (hamster anti-mouse CD3ε; BD Biosciences; catalog no. 550277), followed by an appropriate secondary (goat anti-hamster Alexa Fluor 647 [Invitrogen; catalog no. A21451]), and NeuroTrace 435/455 fluorescent Nissl.

Images were captured on a Zeiss 880 NLO upright microscope, with a 20× objective, and imported into Bitplane Imaris software. Within Imaris, the spots feature was used to represent the cell body of each respective cell of interest, TINs, Bystanders, and T cells, based upon fluorescent staining. X,Y,Z positions from each spot were exported into Matlab to calculate the distances of each T cell to either Bystanders or TINs. T cells within 20 μm of a TIN or Bystander were used for analysis.

### Data processing and differential gene expression analysis, parasite transcripts.

Fastq files were first mapped against the mouse transcriptome (GRCh38 v21; parameters –k 5, –very-sensitive-local), and the nonmapping reads were then mapped to the T. gondii ME49 genome (v44; parameters –k 5 –very-sensitive-local). Raw transcript counts were determined using featureCounts from the Subread package integrating the gff file from ToxoDB and the sorted bam file for each sample. Genes were removed from the analysis if they had ≥20 total mapping reads across the 5 LCM samples obtained from saline-injected mice. Genes were then normalized in two different ways. To determine the relative quantity of parasite-specific reads across all samples, data were transformed using the formula log_2_[1 × 10^6^ × (read counts/total host mapping reads)] where total mapping reads were obtained from Table S1 to generate the log_2_(FPM) value and then filtered based on read counts across samples to include only those genes with at least 1 read in at least 15 of the 25 samples (resulting in 527 genes total). For analysis of the TINs separately from Bystanders and cells from mock-treated control mice, data were included only if they had at least 1 read in 6 of the 25 samples (resulting in 1,927 genes total), and raw counts were uploaded into the DESeq2 package in R and normalized using the “rlog” function. Differential expression between strains was determined using default settings (“results”). These two methods of normalization are why the log_2_(FPM) values are of different scales.

### Data availability.

All raw sequencing data have been deposited in NCBI’s Sequence Read Archive (SRA) and are accessible through BioProject number PRJNA642650. RNA sequencing has been supplied for public availability to ToxoDB (https://toxodb.org/toxo/), which is now a part of VEuPathDB.org.
